# Environmental and Genetic Variables Influencing Mitochondrial Health and Parkinson's Disease Penetrance

**DOI:** 10.1155/2018/8684906

**Published:** 2018-03-07

**Authors:** Alessandra Zanon, Peter P. Pramstaller, Andrew A. Hicks, Irene Pichler

**Affiliations:** ^1^Institute for Biomedicine, Eurac Research, Affiliated Institute of the University of Lübeck, Bolzano, Italy; ^2^Department of Neurology, General Central Hospital, Bolzano, Italy; ^3^Department of Neurology, University of Lübeck, Lübeck, Germany

## Abstract

There is strong evidence that impairment of mitochondrial function plays a key role in the pathogenesis of PD. The two key PD genes related to mitochondrial function are Parkin (*PARK2*) and PINK1 (*PARK6*), and also mutations in several other PD genes, including *SNCA*, *LRRK2*, *DJ1*, *CHCHD2*, and *POLG*, have been shown to induce mitochondrial stress. Many mutations are clearly pathogenic in some patients while carriers of other mutations either do not develop the disease or show a delayed onset, a phenomenon known as reduced penetrance. Indeed, for several mutations in autosomal dominant PD genes, penetrance is markedly reduced, whereas heterozygous carriers of recessive mutations may predispose to PD in a dominant manner, although with highly reduced penetrance, if additional disease modifiers are present. The identification and validation of such modifiers leading to reduced penetrance or increased susceptibility in the case of heterozygous carriers of recessive mutations are relevant for a better understanding of mechanisms contributing to disease onset. We discuss genetic and environmental factors as well as mitochondrial DNA alterations and protein-protein interactions, all involved in mitochondrial function, as potential causes to modify penetrance of mutations in dominant PD genes and to determine manifestation of heterozygous mutations in recessive PD genes.

## 1. Introduction

Parkinson's disease (PD) is the second most common neurodegenerative disease after Alzheimer's disease and is clinically defined as a motor syndrome consisting of levodopa responsive parkinsonism (bradykinesia, tremor, rigidity, and postural instability) and the absence of markers suggestive of other diseases [1]. It is characterized pathologically by loss of dopaminergic (DA) neurons in the *substantia nigra pars compacta* (*SNpc*), loss of DA innervation in the striatum, and the presence of *α*-synuclein positive aggregates (Lewy bodies). PD has an age-related prevalence, affecting around 1% of the population over the age of 65, rising to up to 3% among individuals 80 years of age and older [2]. While most cases are thought to be sporadic, in about 10% of the patients, a genetic cause can be detected, ascribable to mutations in more than a dozen genes [3]. The rare monogenic forms clinically mimic the sporadic form and can thus serve as a disease model for this much more common form of PD. Mutations in several PD causing genes, encoding PINK1 (PTEN-induced serine/threonine kinase 1), Parkin, *α*-synuclein, LRRK2 (leucine-rich repeat kinase 2), DJ1, CHCHD2 (coiled-coil-helix-coiled-coil-helix domain-containing 2), and POLG (mitochondrial DNA polymerase gamma), have been shown to induce mitochondrial dysfunction demonstrating that mitochondrial homeostasis and quality control have a central role in the disease process [4–14]. Several aspects of mitochondrial biology have been described as impaired in different PD models, leading to a defect in electron transport chain enzyme activities, ATP depletion, and generation of reactive oxygen species [15]. Systemic administration of the pesticides rotenone and paraquat as well as the neurotoxicant MPTP (1-methyl-4-phenyl-1,2,3,6-tetrahydropyridine), which are inhibitors of the mitochondrial respiratory chain NADH dehydrogenase (complex I), induces neuropathologic and behavioral changes in rodents similar to human PD [16, 17]. These data from basic science have been replicated in clinical studies showing a 20–30% decrease of mitochondrial complex I activity in the *SNpc* [18, 19], in platelets [20, 21], and in lymphocytes of patients with sporadic PD [22], suggesting a systemic inhibition of complex I activity at least in a subset of the common form of PD. Aging is the biggest risk factor for PD, and during aging mitochondrial function declines, with an accumulation of deletions in the mitochondrial DNA (mtDNA) [23, 24]. In line with these data, mtDNA depletion has been observed in nigral neurons from PD patients [25], but the mechanisms by which these mutations affect biochemical pathways leading to mitochondrial dysfunction are not answered yet. However, mtDNA levels or age-dependent increases in mtDNA mutations may be factors that influence penetrance of nuclear mutations known to act via mitochondrial mechanisms. While the phenomenon of reduced penetrance was initially described in the context of family studies, the advent of next-generation sequencing has revealed an unexpectedly large number of putatively pathogenic mutations in overtly healthy individuals, raising the need for a better understanding of factors influencing penetrance. Indeed, for several mutations in autosomal dominant PD genes penetrance is markedly reduced, whereas heterozygous carriers of mutations in recessive PD genes may also manifest disease or subclinical phenotypes of disease. In the following sections, we will review the evidence supporting the hypothesis that gene-environment, nuclear gene-mtDNA, and protein-protein interactions are able to modify the penetrance of genetic forms of PD.

## 2. Environmental and Genetic Factors and Their Interplay as Modifiers of PD Penetrance

For long time, environmental factors were thought to be the main cause of PD largely due to the epidemic of postencephalitic parkinsonism after the First World War [26]. Furthermore, this hypothesis was additionally supported by the identification of the mitochondrial neurotoxin MPTP in the early 1980s, which causes selective DA neuron degeneration by inhibiting mitochondrial respiratory electron transport chain complex I, leading to a parkinsonian syndrome in rodents, primates, and humans [27, 28]. MPP+, the neurotoxic oxidation product of MPTP, concentrates in DA neurons of the *SNpc* through the dopamine transporter, explaining the selective DA neuronal death [29, 30]. Since then, several epidemiological and experimental studies have evaluated the role of a large number of environmental conditions and agents, including farming and rural life, industrial toxins, heavy metals, smoking, and drinking tea or coffee, in the pathogenesis of PD. Remarkably, a protective association was found for caffeine consumption [31] as well as for cigarette smoking, which could decrease the risk in a dose-dependent manner [32]. On the contrary, epidemiological studies have described the exposure to pesticides, many known to inhibit electron transport chain activity, as an adverse risk factor for PD [33–36]. Importantly, the exposure to toxins like the pesticide rotenone and the herbicide paraquat reduced the activity of mitochondrial respiratory chain complex I and caused neurological defects similar to PD in humans and in animal models [37, 38]. Chronic systemic administration of low doses of rotenone into a rat model induced nigrostriatal cell death and accumulation of proteinaceous inclusions similar to Lewy bodies [39]. However, data collected in another rotenone-based rat model did not support the generation of specific lesions of the *SNpc* suggesting a generalized mitochondrial failure [40]. A mouse model with a deletion of the *Ndufs4* gene, encoding a subunit of complex I, and therefore having reduced complex I activity did not show significant DA neuron loss or motor impairment during lifespan, but showed a reduced amount of dopamine in the brain, increased *α*-synuclein phosphorylation in DA neurons of the *SNpc*, and nonmotor symptoms including impaired cognitive function and increased anxiety-like behavior [41, 42]. These findings suggest that inhibition of complex I activity contributes to dopamine loss and *α*-synuclein pathology and promotes nonmotor symptoms of PD, but it is not sufficient to cause neurodegeneration during aging, suggesting the existence of additional susceptibility factors.

Of note, gene-environment interaction analyses have linked genetic variants in *DAT*/*SLC6A3* likely affecting transport of chemicals into DA neurons to an increased risk for developing PD from exposures to the pesticides paraquat and maneb. Furthermore, variants in the metabolizing enzymes encoded by *PON1* and *NOS1* that may contribute to the nitrosative stress pathway were found to increase susceptibility to PD for organophosphate pesticide exposure. In addition, *ABCB1* gene variants that affect blood-brain barrier transport of chemicals increase organochlorine pesticides effect, and an association between pesticides inhibiting aldehyde dehydrogenase (ALDH), involved in dopamine metabolism, with *ALDH2* gene variants was detected. These genes impact mitochondrial function via oxidative/nitrosative stress pathways and proteasome inhibition [43].

In addition to environmental and genetic risk factors, gene-environment interactions as well as aging as the main risk factors for developing PD, intestinal microbiota have recently emerged as an additional factor able to promote *α*-synuclein-induced motor deficits and microglia activation in the brain. Interestingly, transfer of microbiota from PD patients to *α*-synuclein-overexpressing mice worsened motor impairments compared to microbiota from healthy individuals [44]. In line with these data, several studies have reported differences in the composition of gut microbiota between PD patients and controls [45–47]. While the major interest has been on the influence of such microbiota on the human immune system, there is some evidence that microbial metabolites (e.g., short chain fatty acids) influence mitochondrial function [48]. Relating the specific microbiome-derived metabolites of PD patients to mitochondrial health is an exciting open research question, which will contribute to our knowledge of factors possibly influencing penetrance of PD.

## 3. Mitochondrial DNA Alterations and Reduced Penetrance of Nuclear PD Genes

Human mtDNA is a small, circular 16,569 bp DNA encoding 13 subunits of mitochondrial respiratory chain proteins including seven complex I, one complex III, three complex IV, and two complex V subunits that are essential to the assembly and function of the mitochondrial respiratory chain [49, 50]. Specific mutations in mtDNA have been found in patients with different forms of parkinsonism associated with mitochondrial disorders [51–53], and thus, mtDNA mutations are suspected to contribute to complex I deficiency in PD. Three different types of mtDNA alterations, mtDNA point mutations, mtDNA deletions, and mtDNA copy number have been investigated in postmortem brains or peripheral tissues like blood and skeletal muscle of PD patients. Specific mtDNA regions or the entire mtDNA genome were sequenced in several studies, and numerous point mutations were described [54–60]. However, no specific rare variant linked to PD was identified so far. There is conflicting reports in the literature describing the association between heteroplasmic variants and PD. Coxhead et al. found an increased mutational burden in both the *SNpc* and frontal cortex of sporadic PD patients and a significant overrepresentation of PD cases harboring nonsynonymous heteroplasmic variants in *MTCOX1*, *MTCOX2*, and *MTCYTB* genes in the *SNpc* [61]. These findings could not be replicated in a recent study reporting a high frequency of heteroplasmy in the human brain, which did however not change with age or with PD disease state [60]. Furthermore, several groups have investigated the association of mtDNA haplogroups, aggregations of specific mtDNA variants, with PD. A reduced risk of PD was found for haplogroups J, K, and T, including a variant in the ND3 subunit of complex I, whereas the superhaplogroup HV showed an increased risk of PD [62, 63]. Moreover, not only heteroplasmic point mutations but also heteroplasmic deletions have been investigated, and a high proportion of multiple mtDNA deletions was found to accumulate in the *SNpc* and other brain regions with age, which was significantly more abundant in PD patients [23, 24]. A recent study on human brain tissue convincingly showed a reduction of mtDNA copy number in single neurons of PD patients as compared to controls, which was more pronounced in neurons with severe complex I deficiency [64]. Interestingly, the MitoPark mouse, generated by direct deletion of mitochondrial transcription factor A (TFAM), which is needed for mtDNA replication, in DA neurons, is characterized by a marked deletion of mtDNA, impairment of oxidative phosphorylation, DA neuron degeneration, and motor deficits that mimic human parkinsonism [65]. These data provide evidence for a direct role of mtDNA alterations in mitochondrial function impairments.

As DA neurons are energetically highly demanding, mtDNA copy number or age-dependent increases in heteroplasmy might be factors that influence penetrance of nuclear mutations known also to act via mitochondrial mechanisms (e.g., Parkin and PINK1). Notably, in the mutator mouse model, characterized by the accumulation of multiple deletions in the mtDNA due to a proofreading-deficient form of POLG (the polymerase responsible for mtDNA replication), neuroprotective compensatory mechanisms at the mitochondrial level were observed [66]. After crossing this mouse with the Parkin knockout mouse, which also does not show neurodegeneration, mitochondrial dysfunction and PD pathology became apparent [67]. This study highlights a key role of high levels of mtDNA mutations in DA neurons and a protective role of Parkin, since its loss synergizes with mitochondrial dysfunction resulting in neurodegeneration.

Similar to POLG, mutations in other nuclear genes, like twinkle, a mtDNA helicase required for mtDNA replication and stability, lead to accumulation of mtDNA deletions and have been found to be associated with parkinsonism [68–70].

These studies provide examples for the association of mtDNA alterations, alone or in combination with nuclear gene mutations, with parkinsonism, and point to a role of mtDNA alterations in modifying penetrance of nuclear DNA mutations.

## 4. Protein-Protein Interactions Regulate Mitochondrial Quality Control and Disease Penetrance

By studying rare large families with a clear Mendelian inheritance, several causative genes have been identified, highlighting common intracellular functions involved in the pathogenesis of PD (mitochondria function and quality control (QC), lysosomal and endosomal pathways, synaptic transmission, and vesicle trafficking) [71]. In addition, genome-wide association studies have provided convincing evidence that polymorphic variants in these genes and 43 additional loci contribute to sporadic PD [72, 73]. Similar to other genetically complex diseases, these variants show only moderate effects on PD risk.

The most compelling evidence for the important role of mitochondrial function and QC in the PD pathogenesis has emerged by the elucidation of the function of the *Parkin* and *PINK1* genes during the last decade. Studies in mammalian cells and the model organism *Drosophila* have demonstrated that Parkin, together with PINK1, regulates the degradation of dysfunctional mitochondria by the autophagy-lysosomal pathway, a process known as mitophagy [8, 74–77]. Other PD-linked proteins, like FBXO7 and VPS13C, might contribute to mitochondrial QC. An interaction of FBXO7 with PINK1 and Parkin contributes to mitochondrial maintenance through Parkin/PINK1-mediated mitophagy [78], and loss of function related to VPS13C exacerbates mitochondrial vulnerability to stress and increases PINK1/Parkin-dependent mitophagy [79].

Through direct or indirect interactions of Parkin/PINK1 with the fusion factors Mfn1, Mfn2, and OPA1 and the fission factor Drp1, these proteins regulate mitochondrial dynamics, which is linked to the maintenance of mitochondrial function [80–83]. Furthermore, Parkin and PINK1 have been implicated in another, autophagy-independent, mitochondrial QC mechanism, which is the transport of specific protein cargo as membrane-derived vesicles (MDVs) to the lysosomes. The formation of MDVs is stimulated as an early response to oxidative stress, and damaged mitochondrial regions are excised and transported to the endolysosomal compartments for degradation [84, 85]. In addition to degradative pathways, Parkin and PINK1 proteins play a crucial role in the regulation of mitochondrial biogenesis through the degradation of the Parkin substrate PARIS, which acts as a transcriptional repressor of peroxisome proliferator-activated receptor gamma coactivator-1 alpha (PGC-1*α*), a transcriptional coactivator and master regulator of mitochondrial biogenesis [86, 87]. Moreover, an involvement of both Parkin and PINK1 in mitochondrial trafficking and QC has been suggested by reporting their association to Miro/Milton/dynein complex [88, 89]. Miro is an outer mitochondrial membrane protein that anchors mitochondria to microtubule motors. As a consequence of Miro phosphorylation mediated by PINK1, Miro is ubiquitylated by Parkin and degraded by the proteasome. This leads to blockage of mitochondrial motility, which facilitates the isolation and sequestration of damaged mitochondria for degradation [88]. For example, an increase in anterograde transport was shown in axons of pink1 knockdown *Drosophila* [90], while mitochondrial trafficking was blocked by overexpression of both Parkin and Pink1 in *Drosophila* and in mammalian cells [88, 91]. More recently, it has been shown that also LRRK2 promotes Miro removal. The pathogenic G2019S mutation disrupts this function, delaying the motility blockage of damaged mitochondria and consequently slowing the initiation of mitophagy [92]. Remarkably, Miro degradation and reduced mitochondrial motility were also detected in sporadic PD patients, pointing to Miro and its interaction with PD-related proteins as a common molecular mechanism in different forms of PD. Recently, we have identified Stomatin-like protein 2 (SLP-2) as a novel Parkin interactor that upon overexpression rescued mitochondrial dysfunction of Parkin-deficient neuronal cells. Double knockdown flies showed a genetic interaction between Parkin and SLP-2, and SLP-2 transgenic flies attenuated loss of DA neurons, mitochondrial network structure, and flight and motor dysfunctions. This interaction might promote optimal activity of mitochondrial respiratory chain complex I and mitochondrial integrity in induced pluripotent stem cell-derived neurons and *Drosophila* [93]. SLP-2 was described as part of a new mitochondrial protein complex named SPY, which regulates proteolysis of PINK1 by the mitochondrial protease PARL linking SLP-2 to both Parkin and PINK1 [83].

Interactions between different PD-causing proteins and/or their binding partners involved in mitochondrial QC might influence disease penetrance as basal and stress-evoked QC mechanisms might fail during ageing and/or the presence of mutations in genes encoding proteins involved in these pathways. The effects of the resulting mitotoxicity are deleterious and ultimately lead to degenerative processes specifically in vulnerable terminally differentiated neurons.

## 5. Conclusions

**Figure 1:**
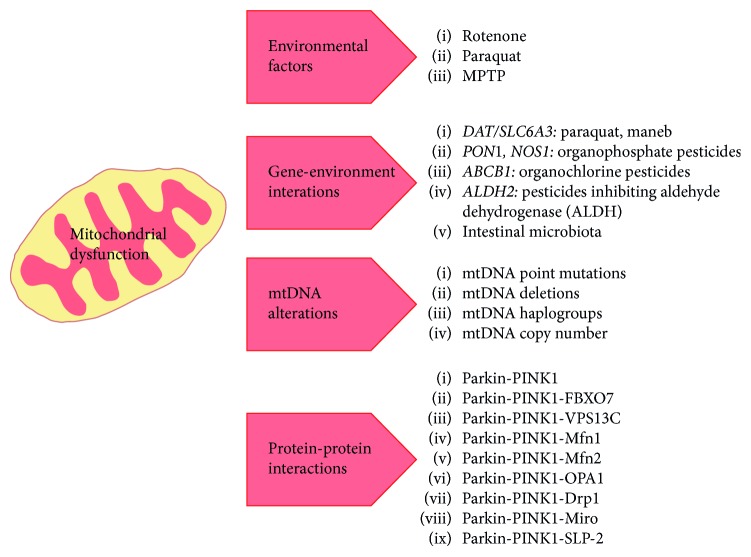
Modifying factors possibly underlying nonpenetrant or highly reduced penetrant forms of PD.

About 10% of all PD patients suffer from a monogenic form where autosomal dominant or recessive mutations in single genes are causative. Autosomal dominant PD is mostly represented by mutations in *SNCA*, coding for *α*-synuclein, and *LRRK2* and recessive PD by mutations in *PINK1* and *Parkin*. Many of these mutations, particularly in the autosomal dominant forms, are only imperfectly penetrant or show a delay in the age at onset. Notably, heterozygous mutations in *Parkin* and *PINK1* are relatively frequent (about 8% of all PD patients screened for *Parkin* mutations [94]) and may predispose to PD or subclinical phenotypes with highly reduced penetrance [95], although this remains controversial [96]. This reminds however mutations in the *GBA* gene (encoding the lysosomal enzyme glucocerebrosidase), which in a homozygous fashion, are responsible for the lysosomal storage disorder Gaucher's disease, while heterozygous mutations in this gene have been established as well-validated risk factors for PD [97, 98]. The vast majority of PD patients are thought to be sporadic with a genetically complex form. In fact, more than 40 loci have been identified that are able to increase PD risk with however only modest to moderate effect size estimates (ORs ranging from 1.1 to 1) [72]. Given the high rate of reduced penetrance in PD, finding factors responsible for an incomplete or delayed disease onset and understanding the underlying mechanisms is important as they might be able to protect from developing the disease or delay the disease onset. We are highlighting environmental and genetic modifiers as well as mtDNA alterations and protein-protein interactions as possible factors underlying nonpenetrant or highly reduced penetrant forms of PD (Figure 1). These modifiers are all involved in the maintenance of mitochondrial function and may result in neuroprotection despite the presence of mutations causing PD in many instances. Validation of such modifiers would have high translational potential for the identification of early diagnosis and novel therapeutic options to halt the disease progression.
